# Seasonal influenza risk in hospital healthcare workers is more strongly associated with household than occupational exposures: results from a prospective cohort study in Berlin, Germany, 2006/07

**DOI:** 10.1186/1471-2334-10-8

**Published:** 2010-01-12

**Authors:** Chris J Williams, Brunhilde Schweiger, Genia Diner, Frank Gerlach, Frank Haaman, Gérard Krause, Albert Nienhaus, Udo Buchholz

**Affiliations:** 1 Department for Infectious Disease Epidemiology, Robert Koch Institute, Berlin, Germany; 2 European Programme for Intervention Epidemiology Training, ECDC, Stockholm, Sweden; 3 Vivantes Healthcare, Berlin, Germany; 4 Berufsgenossenschaft für Gesundheitsdienst und Wohlfahrtswesen, Hamburg, Germany

## Abstract

**Background:**

Influenza immunisation for healthcare workers is encouraged to protect their often vulnerable patients but also due to a perceived higher risk for influenza. We aimed to compare the risk of influenza infection in healthcare workers in acute hospital care with that in non-healthcare workers over the same season.

**Methods:**

We conducted a prospective, multicentre cohort study during the 2006/07 influenza season in Berlin, Germany. Recruited participants gave serum samples before and after the season, and completed questionnaires to determine their relevant exposures and possible confounding factors. The main outcome measure was serologically confirmed influenza infection (SCII), defined as a fourfold or greater rise in haemagglutination inhibition antibody titres to a circulating strain of influenza (with post-season titre at least 1:40).

Weekly mobile phone text messages were used to prompt participants to report respiratory illnesses during the influenza season. A logistic regression model was used to assess the influence of potential risk factors.

**Results:**

We recruited 250 hospital healthcare workers (mean age 35.7 years) and 486 non-healthcare workers (mean age 39.2 years) from administrative centres, blood donors and colleges.

Overall SCII attack rate was 10.6%. Being a healthcare worker was not a risk factor for SCII (relative risk 1.1, p = 0.70). The final multivariate model had three significant factors: living with children (odds ratio [OR] 3.7, p = 0.005), immunization (OR 0.50, p = 0.02), and - among persons living in households without children - ownership of a car (OR 3.0, p = 0.02). Living with three or more children (OR 13.8, p < 0.01) was a greater risk than living with one or two children (OR 5.3, p = 0.02). 30% of participants with SCII reported no respiratory illness. Healthcare workers were at slightly higher risk of reporting any respiratory infection than controls (adjusted OR 1.3, p = 0.04, n = 850).

**Conclusions:**

Our results suggest that healthcare workers in hospitals do not have a higher risk of influenza than non-healthcare workers, although their risk of any respiratory infection is slightly raised. Household contacts seem to be more important than exposure to patients. Car ownership is a surprise finding which needs further exploration. Asymptomatic infections are common, accounting for around a third of serologically confirmed infections.

## Background

The German standing commission for immunisation, along with other authorities [1-3] currently recommends that healthcare workers (HCWs) be vaccinated against seasonal influenza. Two reasons are cited: firstly that HCWs can be a source of infection for vulnerable people under their care [1-3], and secondly that HCWs are at increased risk for contracting influenza [1]. Vaccination of HCWs should theoretically reduce the risk of influenza infection both in themselves and their patients. There is evidence to support the first reason for vaccination, the protection of patients. HCWs can transmit influenza to those under their care during both outbreaks and non-outbreak situations [4-7]. Low vaccination rates in HCWs have been associated with nosocomial outbreaks [4,8], and higher vaccination rates with reduced nosocomial influenza incidences [7].

HCWs may transmit influenza to those under their care, but is there evidence that their occupational exposures (to patients, relatives, colleagues and the hospital environment) confer an increased risk of influenza compared to the general population? Influenza serological attack rates in HCWs of 23% (single season, [9]) and 14% (an average of two seasons, [10]) have been documented. However, as serological attack rates of influenza may vary considerably from season to season as well as from location to location, without the inclusion of a comparison group of non-HCWs neither study could demonstrate an increased risk.

Another aspect of influenza in HCWs is that many HCWs argue that they withdraw from work when they become ill with influenza-like illness to reduce their risk of transmitting influenza to their patients. However, not all serologically diagnosed influenza infections experience an influenza-like illness, and a proportion will be asymptomatic. The frequency of asymptomatic influenza infection in HCWs has been assessed in volunteer studies (33%, [11]), a cohort study (28%, [9]) and one randomised controlled study (42%, [10]).

The objective of this study was to address the question of whether HCWs in the acute care hospital setting have a higher risk of serologically confirmed influenza infections (SCII) than non-HCWs, and to assess the proportion of individuals with SCII who experience either any respiratory symptoms or an influenza-like illness.

## Methods

We conducted our study in people living or working in Berlin during the influenza season of 2006/07, using a multicentre, prospective cohort design.

There were 11 study sites: three hospitals, two administrative centres (the Robert Koch Institute and Vivantes Healthcare administrative centre), four blood-donation centres and two colleges. HCWs and some non-HCWs were sought from the hospitals, but only non-HCWs were sought from all other sites.

We recruited participants through occupational health services in the hospitals and one administrative centre; through direct recruitment at blood donation centres; and through active recruitment during site visits at the other study sites (non-healthcare workers from one administrative centre and two colleges).

On recruitment before the influenza season, consenting participants gave a single serum sample and completed an exposure questionnaire. Details recorded included age, sex, type of employment, risk factors for influenza, smoking status, and vaccination status. Where participants had been vaccinated fewer than 14 days before the sample was taken, or were found to have been vaccinated shortly after the initial sample was taken, a second sample, taken at least 14 days after vaccination, was sought through direct recall or through occupational health.

Blood samples were refrigerated, then transported within three days to the national reference laboratory for influenza at the Robert Koch Institute in Berlin, where they were centrifuged and frozen.

### Inclusion and exclusion criteria

HCW in our context were defined as people working on a daily basis with unwell patients in an acute hospital setting, including nurses (trainee and qualified) and doctors. Non-HCW were those working or studying at the study sites, or attending the blood donation centres, who did not fit the definition of HCW.

Exclusions (applied to both HCWs and non-HCWs) were: people with patient contact in the community (such as community doctors and nurses, dentists, and pharmacists); people working in care homes; laboratory workers who had contact with respiratory samples or with influenza virus; people who planned to be away from Berlin for more than two weeks during the projected season (January to April); and people who did not wish to be contacted weekly by mobile phone Short Message System (SMS) or email.

For the analysis of serologically-confirmed infections, we excluded vaccinated individuals where the baseline serum sample was taken fewer than 14 days after vaccination, or where a later second sample was not obtainable (see recruitment above). These participants were excluded from the serological analyses as any titre rise could have been caused by vaccination and not infection.

Because pigs can carry influenza viruses, and participants with pig contact were associated with SCII, participants reporting contact with pigs were excluded.

### Active surveillance for respiratory infections during influenza season

In order to document weekly occurrences of respiratory infections during the influenza season, we contacted all participants weekly through SMS or through email, asking them if they had experienced a "new" respiratory infection during the previous 7 days. Where participants answered "yes", they were contacted by telephone and details of their infection were obtained using an illness questionnaire.

Weekly surveillance for respiratory infections covered the period from January 13, 2007 to March 30, 2007, a period chosen to coincide with the influenza season. This strategy was employed in order to maximise the predictive value of a positive answer.

### Postseason follow-up

After the influenza season, participants were recalled by SMS or email. They gave a second serum sample and completed a further questionnaire, including repeat questions on vaccination status and employment type; number of patient contacts on a typical day between January 13 and April 6, 2007; broad age classification of patient contacts (adults (>17 years) and children (<18 years); clinical specialty and usage of facemasks (for HCWs); daily professional and household contacts; use of public transport and car ownership; contact with pigs (for veterinary students); and vaccination in previous years. Participants were asked again if they had had respiratory infections over the period January 13, 2007 to 6^th ^April 2007, extending the period of surveillance to one week after the last weekly SMS was sent.

Contact was defined as either touching or having a two way conversation with someone close by, or (for patient contact only) examining or giving care to a patient. Participants were asked to estimate the number of contacts made during a typical day in household and work settings.

### Laboratory investigations

The paired blood samples were defrosted and antibody titres were determined on the same day using the haemagglutination inhibition test to determine infection in any of the two A subtypes or the two B lineages. We tested for antibody to the following strains:

A/H1: A/New Caledonia/20/1999 (H1N1)

A/H3: A/Wisconsin/67/2005 (H3N2), and A/California/07/2004 (H3N2)

B, Victoria lineage: B/Malaysia/2506/2004

B, Yamagata lineage: B/Jiangsu/10/2003.

Titres of below 10 were assigned the value of 5 in order to allow calculation of the titre rise. SCII was defined as a fourfold or greater titre rise between pre- and post-season samples, with a postseason titre of at least 40. As A/Wisconsin/67/2005 (H3N2) and A/California/07/2004 (H3N2) were closely related a titre rise to either of these strains was considered as a single SCII due to A/H3N2.

### Outcomes

The primary outcome was evidence of SCII by any of the above strains.

The clinical outcomes were influenza-like-illness (ILI), defined as an illness with an acute onset, self-reported fever, cough, and head or body pains; and acute respiratory infection (ARI), defined as any reported infection with coryza (nasal discharge) or cough. Clinical outcomes were based only on completed illness questionnaires or postseason illness reports, not on SMS or email replies, the latter being used only as the prompt for collection of illness data.

Where illness was reported over more than one week, symptoms for each week were combined to produce a single illness episode. To produce an epidemic curve of SCII with any ILI, or in its absence, another ARI, the respective dates of illness onset were plotted. Where more than one illness episode was reported, we used the onset date of the episode closest to the peak, on the assumption that this episode was the most likely to be due to influenza virus.

### Statistical analysis

We undertook bivariate analyses for all binary exposure variables and calculated risk ratios (RR), their 95% confidence interval and p-values. For the comparison of the exposure groups (HCW vs. non-HCW) continuous variables were analysed using the Kruskal-Wallis test. For the analysis of the association with SCII continuous variables were explored by grouping them in categories. We regarded a p-value of less than 0.05 as statistically significant. We then constructed a multivariate logistic regression model (*logistic *command, STATA [StataCorp. 2007. Stata statistical software: Release 10. College Station, TX: Statacorp LP]) using variables which were associated with the outcome with a p-value of less than 0.1 in the bivariate analysis. Variables with p values between 0.1 and 0.2, along with healthcare worker status, were also tested in the final model.

In order to determine whether the site of recruitment had any group-level effects on the model, we constructed a random-effects logistic regression model with the same variables as the standard model plus study site as the grouping variable, and a likelihood ratio test for the proportion of variance attributable to the group level (rho) was performed (*xtlogit*, STATA).

### Data protection and ethical approval

The data protection protocol was approved by both state and national data protection offices in Berlin, and shared with all study partners. Ethical approval for the study was obtained from the University of Berlin, faculty of medicine (Charité) ethics committee.

## Results

### Recruitment and follow-up

**Figure 1:**
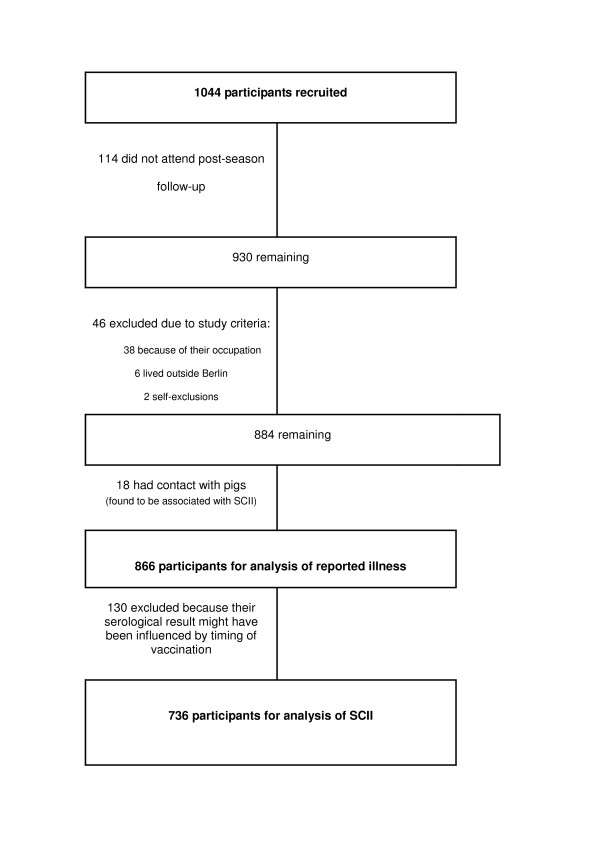
**Exclusions and losses of study participants**. SCII = serologically confirmed influenza infection.

We recruited 1044 participants, of which 736 (71%) were included in the analysis with SCII as outcome, and 866 (93%) for the clinical outcome analysis (Figure 1).

Those not included in the study were of younger age (38 vs 34, p < 0.01 Kruskall-Wallis) and were more likely to be HCW at recruitment (42% vs 34%, p < 0.01), but there were no significant differences in sex, current immunisation, smoking status, car ownership or use of public transport in excluded versus non-excluded participants.

### Comparison of study participants by healthcare worker status

Of the 736 participants included in the analysis with the outcome SCII 250 (34%) were HCWs and 486 (66%) were non-HCWs. Most participants (71%) were female. The age distribution was bimodal, with peaks in the age groups 20-29 years and 40-49 years both in HCWs and non-HCWs.

Of the 250 healthcare workers, 41 (16%) were doctors, 97 (39%) were trainee nurses and 112 (45%) were qualified nurses. Of the 486 non-healthcare workers, 178 (36%) were administrative or information technology staff, 107 (22%) scientific staff, 45 (9%) students, 6 (1%) teaching or lecturing staff, 25 (5%) in manual or technical roles, 21 (4%) in retail or service, 9 (2%) veterinary staff, and 7 (1%) in other occupations. In 88/486 (18%) the occupation was not stated or unclear.

Table 1 compares study characteristics in the exposure group (HCW) and control group (non-HCW). HCWs were significantly younger than controls (35.7 versus 39.2, Kruskal-Wallis test, p < 0.01). They were also significantly more likely to be current smokers, female, vaccinated in the current season, and to own a car.

**Table 1:** Characteristics of study participants, by healthcare worker status

Exposure (n = 736 unless otherwise specified)	Healthcare workers (N = 250)	Non-Healthcare workers (N = 486)	P (chi-squared, unless otherwise specified)
Age, mean (years)	35.7	39.2	P < 0.001 (Kruskal-Wallis)
Male sex (%)	57 (22.8%)	160 (32.9%)	P < 0.001
			
Immunisation in current season (2006/07) (n = 736)	104 (41.6%)	153 (31.5%)	0.006
			
Immunised 2005/6 (n = 728)	103 (41.7%)	184 (38.3%)	0.37
Immunised 2004/5 (n = 712)	77 (32.1%)	162 (34.3%)	0.55
Immunised 2003/4 (n = 696)	53 (22.7%)	132 (28.6%)	0.10
Current smoker at recruitment (n = 726)	99 (40.6%)	125 (25.9%)	P < 0.01
Children at home	87 (34.8%)	144 (29.6%)	0.15
Patient contacts (per typical day)	30.8	Not applicable	
			
Contacts at work (per typical day, excluding patients), mean (n = 730)	14.3	13.8	0.021 (Kruskal-Wallis)
Contacts at home (per typical day), mean	2.7	3.5	P < 0.001 (Kruskal-Wallis)
Car owner (n = 727)	199 (80.6%)	352 (73.3%)	0.03
Regular use of public transport (n = 733)	128 (51.6%)	303 (62.5%)	0.005
Preseasonal titer of <40 against A/California/07/2004 (H3N2) among non-vaccinated	97 (69.7%)	269 (79.1%)	0.03

Healthcare workers had significantly more non-patient contacts at work (mean 14.3 versus 13.8, p = 0.021) but fewer contacts at home (mean 2.7 versus 3.5, P < 0.01) There were no significant differences in immunisation in the previous three seasons, or in household exposure to children. HCWs were significantly less likely to have a titer of <40 against A/California/07/2004, the A/H3N2-strain of the previous season.

### Serologically confirmed influenza infection and ILI/ARI

In total there were 82 titer rises among 78 participants. Of these, 13 (16%) were due to A/New Caledonia/20/1999 (H1N1), 64 (78%) due to either of the two A/H3N2-strains tested, two (2%) due to B/Malaysia/2506/2004 and three (4%) due to B/Jiangsu/10/2003. There were four double-infections, three with H3N2/H1N1 co-infections and one with titre rises to both B strains.

Table 2 shows SCII and reported infections by healthcare worker status. The overall attack rate for SCII was 10.6% (78/736). Of the 78 people with evidence of SCII, 23 (30%) reported neither ARI nor ILI, 33 (42%) reported at least one ARI but no ILI, and 22 (28%) reported at least one ILI.

**Table 2:** Serologically confirmed influenza infections (SCII) by healthcare worker status and reported respiratory illness (n = 736)

		Number with SCII (% in illness group)		
		
Reported illness	N	Healthcare workers	Non-healthcare workers	All
**No reported illness**	421	8 (5.9)	15 (5.3)	23 (5.5)
**Acute respiratory infection (excluding ILI)**	231	11 (13.9)	22 (14.5)	33 (14.3)
**Influenza-like illness (ILI)**	84	9 (25.7)	13 (26.5)	22 (26.2)

**Total**	736	28 (11.2)	50 (10.3)	78 (10.6)

### Bivariate analysis: risk factors for SCII

HCWs did not have a significantly higher risk of SCII than non-HCWs (RR 1.09, p = 0.70). In addition, neither working as a nurse (RR = 0.94, p = 0.82) nor as a doctor was significant (RR = 1.76, p = 0.13). There was also no significant difference in the risk of SCII between HCWs and controls after stratification by vaccination status, car ownership, having children, or regular use of public transport.

**Figure 2:**
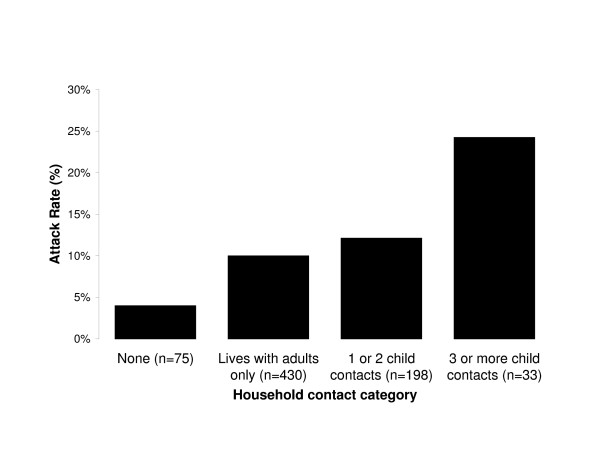
**Unadjusted attack rates for serological influenza infection by category of household contacts (n = 736)**.

There were three exposures with p-values below 0.1: vaccination, having a car in the household and having three or more children at home (Table 3). Household contacts increased the unadjusted risk of SCII (Figure 2). Attack rates were lowest in those living alone (4%), intermediate in those living with adults but no children (10%) or one or two children (12%), and highest in those with three or more children in the household (24%). In a stratified analysis, the effect of car ownership was significant when participants did not live with children (RR = 2.77, p = 0.01), but not for participants who lived in a household with children (RR = 0.98, p = 1.00).

**Table 3:** Attack rates (AR) and relative risks for persons with serologically confirmed influenza infection (SCII) as outcome, by exposure (n = 736), sorted by p-value

	Exposed			Unexposed					
					
Exposure	SCII	Total	AR%	SCII	Total	AR%	Relative risk	95% confidence interval	P-value
Household car ownership	66	551	12.0	10	176	6	2.11	1.11-4.01	**0.02**
Immunisation in current season	18	257	7.0	60	479	13	0.56	0.34-0.93	**0.02**
Household contacts									
*Lives alone*	3	75	4.0	-	-	-	**Ref**	-	-
*Lives with adults*	43	430	10.0	-	-	-	2.50	0.80-7.85	0.13
*1 or 2 children*	24	198	12.1	-	-	-	3.03	0.94-9.77	0.07
*3 or more children*	8	33	24.2	-	-	-	6.06	1.72-21.41	**<0.01**
7 or more daily child patient contacts	8	45	17.8	70	691	10.13	1.75	0.90-3.42	0.11
Doctor	7	39	18.0	71	697	10.19	1.76	0.87-3.57	0.13
Being employed	70	685	10.2	8	51	15.69	0.65	0.33-1.28	0.22
Current smoker (at recruitment)	28	224	12.5	50	502	9.96	1.25	0.81-1.94	0.31
Male sex	25	217	11.5	53	519	10.21	1.13	0.72-1.77	0.60
Working 30+ hours per week	60	581	10.3	18	155	11.61	0.89	0.54-1.46	0.64
Healthcare worker	28	250	11.2	50	486	10.29	1.09	0.70-1.68	0.70
Regular public transport use	47	431	10.9	31	302	10.26	1.06	0.69-1.63	0.78
Nurse (qualified or trainee)	20	206	9.7	50	486	10.29	0.94	0.58-1.54	0.82
Age 16-24 years	18	177	10.2	60	559	10.7	0.95	0.57-1.56	0.83
Age 25-49 years	44	392	11.2	34	344	9.9	1.14	0.74-1.73	0.56
Age 50+ years	16	167	9.6	62	569	10.9	0.88	0.52-1.48	0.78

### Multivariate model

Variables significantly associated with SCII were the type of household contact environment, car ownership, and vaccination status (Table 4). Living with children was associated with an increased risk of SCII, with an overall odds ratio of 3.7 (p < 0.01, separate model without stratification of household contacts). Three or more children in the household (OR13.8, p < 0.01) were a greater risk than one or two children (OR 5.3, p = 0.02).

**Table 4:** Logistic regression model for persons with serologically confirmed influenza infection as outcome (n = 727)

Exposure	Number exposed	**Odds Ratio**^**1**^	P-value	95% confidence interval
**Immunisation**	257	0.50	0.02	0.29-0.88
**Household contacts**				
Lives alone	75	ref	-	-
Lives with adults	430	2.0	0.28	0.58-6.70
1 or 2 children	198	5.3	0.02	1.33-21.12
3 or more children	33	13.8	0.001	2.97-64.27
**Lives without children and has a car**	352	3.0	0.02	1.21-7.25

1 - adjusted for all other variables in model

Household car ownership was a significant risk factor only among persons living in households without children and had an odds ratio for SCII of 3.0 (p = 0.02), while owning a car in households with children was not statistically significant (OR = 0.95; p = 0.94) and so was not included in the final model.

Immunisation against influenza was associated with an OR of 0.50 (p = 0.02). Vaccine effectiveness against SCII was therefore 50% (95%CI 12-71%). Effectiveness against SCII with a reported ILI was higher at 73% (95%CI 6-92%; p = 0.04).

Other variables were added to the multivariate model, but were found not to be significant at the p < 0.2 level. These were being a doctor; having 7 or more child patient contacts; and being a healthcare worker. Addition of a group-level variance term for recruitment site to the random-effects model was not statistically significant (p(rho = 0) > = 0.498).

### Risk factors for any ARI

The four variables with a p-value < 0.2 in the bivariate analysis were age below 51 (p = 0.02), female sex (p = 0.03), HCW (p = 0.03) and smoking (p = 0.12). In the multivariate model (n = 850) all except female sex (p = 0.054) were significant, and the effect of smoking became statistically significant. Age below 51 (OR 1.44, p = 0.04), female sex (OR 1.36, p = 0.05) and HCW status (OR 1.34, p = 0.04) had an OR above 1, and smoking had an OR below 1 (OR 0.72, p = 0.04).

### Temporal progression of influenza cases

**Figure 3:**
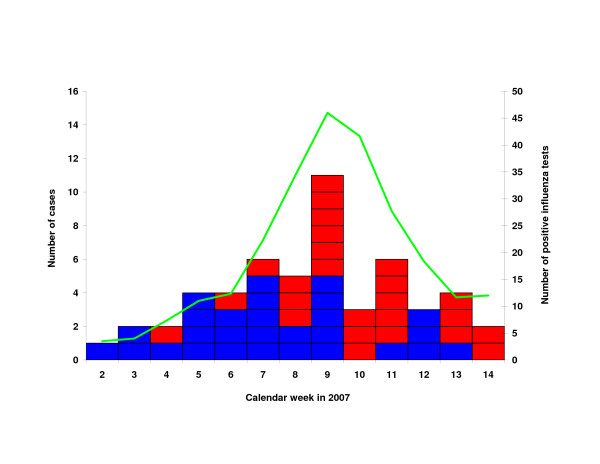
**Cases of serologically confirmed influenza infection reporting acute respiratory illness (ARI; blue) and influenza-like illness (ILI; red), by date of onset of reported illness (n = 53)**. Green line (on secondary y-axis): smoothed line of influenza cases in Berlin identified at the national reference laboratory for influenza from samples collected within the German sentinel surveillance system [12].

Figure 3 shows the distribution of dates of onset for all SCII where an episode of ARI or ILI was reported. The peak seen in week 9 corresponded with the peak number of positive influenza tests from Berlin patients performed at the national reference laboratory for influenza, from patient samples collected through the German influenza sentinel surveillance system [12].

## Discussion

We found no significant association between being a healthcare worker in acute hospital care and serogically confirmed influenza infection. Instead we identified household contacts, in particular children, and car ownership, as important risk factors for SCII. 30% of participants with SCII reported no symptoms, and only around one-quarter of those reporting influenza-like illness also had SCII.

We did not demonstrate a strongly increased risk of influenza infection in HCWs in acute hospital care. As HCWs do appear to have an increased risk for ARI (OR = 1.3) it would be plausible to expect an effect of similar order for influenza infections. Although study limitations might have led to a failure to detect a true increase, it is unlikely that we have missed a large difference in the risk of influenza.

Why might HCWs in hospital not have a higher risk of influenza? The prevalence of infectious influenza in patients may have been low, either due to the absence of influenza patients or because patients admitted later in the course of illness might have been less infectious. Also, infection control measures such as use of personal protective equipment and individual or cohort isolation might have reduced the risks of infection in HCWs. Lastly, prior immunity may have played a role. HCWs were less likely than non-HCWs to be susceptible (preseason titer of less than 40) to A/California/07/2004 (H3N2), which was the A/H3N2 strain of the previous season and related to A/Wisconsin/67/2005 (H3N2), the dominant strain in the 2006/07 season.

As most influenza patients admitted to hospitals are children or older people, one might expect to find an increased risk for SCII in HCWs who work with these patient groups. Our finding of an elevated odds ratio for HCWs with more frequent contact with child patients, albeit not significant in this setting, would be consistent with this supposition. Another study focusing on this hypothesis is needed to investigate it further.

Household contacts, and in particular children in the home, were the main significant risk factors identified in this study. This finding is similar to that in another droplet-transmitted infection, meningitis, where household contacts constitute by far the most important risk group [13]. We found a strong dose-response relationship for child household contacts. The role of children as the main sources of influenza transmission has been suggested in several studies [14-17]. Adult household contacts may play a role analogous, but possibly less marked, to that of children.

Whilst public transport usage was not associated with SCII, car ownership was a significant risk factor, albeit only in households without children. This was unexpected, as car ownership was only included as a counterpart question to public transport usage. For this reason, further details on passengers, frequency or duration of car use were not obtained in the study.

Possession of a car has not previously been recognised as a risk factor for influenza acquisition. However, sharing a car does involve prolonged close contact in an enclosed space and has been linked to transmission of one airborne pathogen [18]. In one observational study on risk factors for SARS in China "using a taxi more than once a week" was identified as independent risk factor for SARS infection with an increased odds ratio close to significance (p = 0.07). Furthermore, this variable was kept in the multivariate model while "riding a bus" and "taking the subway" (who were significant in bivariate analysis) were not [19]. Ownership of a car may also be a marker for having a greater number of social contacts, perhaps including children. Car users are also exposed to air pollution from other vehicles, and this may predispose users to respiratory infections. Despite the unexpected nature of this finding, it is possible that car usage is indeed a risk factor and should be explored through further studies.

We did not find an increased risk of influenza in the 58% of participants who were regular users of public transport. Public transport usage has been cited as a possible risk factor for influenza infection, particularly in the context of pandemic planning [20]. In a recent international survey of precautionary behaviour for pandemic influenza, 75% of respondents said that they would avoid public transport [21]. Both rail and air travel have been associated with respiratory infection transmission [22,23]. Whilst it is possible to contract influenza on public transport, our results suggest that, at least in Berlin, using public transport does not increase a person's risk of influenza. In Berlin, crowding on public transport is infrequent and not extreme, so this result may not be generalisable to cities with different conditions.

Vaccine uptake was relatively high both in HCWs (42%) and non-HCWs (32%). Reasons for this high HCW uptake include the participation of a hospital from former East Germany where vaccination uptake in hospitals is still found to be higher than in West German hospitals. In non-HCWs, the participation of a federal public health institute where influenza vaccination is freely offered to employees may account for the high vaccination rate. High vaccination uptake limited the number of SCII and reduced the study power. Vaccination effectiveness was 50% against SCII and reached 73% for a more severe outcome, SCII with reported ILI.

Only 28% of participants with SCII reported an ILI, the majority reporting no symptoms or more minor respiratory illness. The proportion of asymptomatic infections (30% or more) was substantial and similar to that reported by Elder (28%,[9] and consistent with an estimated proportion of 33% obtained from pooled challenge studies [10]. In addition, only 26% of persons with an ILI had evidence of an influenza infection. Thus, the ILI-syndrome was a poor marker for influenza infections. Unvaccinated HCWs who intend to withdraw from work when they become ill with influenza-typical symptoms may overlook many (symptomatic or asymptomatic) influenza infections, putting patients at unnecessary risk.

The association of HCW status and ARI may reflect the fact that HCWs communicate with or care for a large number of people including patients, colleagues, and relatives, and are thus exposed to a wide variety of respiratory pathogens. The reduced risk for ARI in people aged over 50 years might be explained by their cumulative immune experience or a lower contact rate. The apparent protective effect of smoking could be due to lower ascertainment of ARI in this group, where a background of smoking-related symptoms may have masked the onset of an additional, mild infectious respiratory illness. Conversely, respiratory symptoms due to non-infectious causes may be over-reported in non-smokers.

What significance does this study have for the current H1N1v pandemic? As pandemic influenza should result in a higher attack rate than seasonal influenza, the prevalence of influenza in hospital inpatients and staff is likely to be higher than for seasonal influenza, so increasing the risk of exposure and infection for HCWs compared to that described here. For the same reasons, the risk in HCWs and non-HCWs from their household contacts would also be higher than for seasonal influenza. Therefore even if a repeat study during pandemic conditions identified a significant occupational risk for HCWs, household exposures might still be more strongly associated with influenza infection.

This study was subject to a number of limitations. As recruiting at the hospital and one administrative study site was done through the occupational health departments, only a small proportion (fewer than 10%) of the HCWs at these sites were enrolled in the study. Selection bias could have concealed any actual relationship between HCWs status and influenza infection, if HCWs with a lower risk of influenza than their non-recruited colleagues were to have enrolled, or if non-HCWs with a higher risk of influenza enrolled. It is possible that HCWs with a higher number of patient contacts and, therefore in theory a higher risk of influenza, would be less likely to participate due to pressure of work. Alternatively, HCWs who were concerned about their higher risk of influenza may have protected themselves better and may have been more likely than other HCWs to participate, which would tend to decrease the relative risk in this group.

Loss of recruited participants, mostly due to losses to follow up (despite repeated attempts to contact) and to the timing of vaccination, reduced the effective power of the study and may have worsened selection bias, although excluded participants did not differ significantly with respect to variables found to influence the outcome.

Although there were differences between the two comparison groups with respect to sex, recent immunisation, smoking and use of public transport, the logistic regression methods should have adjusted for these where they had an effect on the outcome (in particular immunisation). The lower average age and higher proportion of females in the HCW group is likely to be due to recruitment among trainee nurses in the larger hospitals.

Serological testing for influenza infection alone, rather than molecular testing methods, may have underestimated the true number of influenza infections [24]. This, along with the relatively high vaccination uptake, will have reduced the number of SCII and thus the effective study power. With an attack rate of 10% in non-healthcare workers, the sample size of 736 analysed would not have been sufficient to detect a relative risk below 1.8 (80% power, 5% significance).

We explored the possibility of influenza clustering due to site-level group influences or localized influenza outbreaks. Analysis of symptom onset dates revealed no evidence of separate site-specific outbreaks, but instead the epidemic curves at each study site followed the overall trend. In addition the multilevel analysis suggested that there were no significant group-level influences on the outcome.

## Conclusions

Our study results suggest that HCWs in an acute hospital care setting are at no higher risk of influenza than the general public, or that if they are, the increased risk is modest. Household contacts, particularly children, play an important role with individuals with three or more children being at highest risk. Use of public transport does not increase the risk of influenza, whereas (in the absence of household children) car ownership does seem to significantly increase the risk for influenza infection, although the mechanism is unclear. Further research would help to clarify the role of household contacts of different age groups, the relevance of car ownership, and whether subgroups of hospital HCWs - or HCWs in other settings, such as in primary care, - are at increased risk for influenza infection. Finally, the ILI-syndrome is a poor marker for influenza infection, suggesting that HCWs cannot rely on this syndrome if they wish to confidently protect their patients but, should instead be vaccinated.

## Authors' contributions

CW, under the supervision of UB, led the study design, conducted the analysis and drafted and updated the manuscript. CW had full access to all the data in the study and takes responsibility for the integrity of the data and the accuracy of the data analysis.

BS supervised laboratory testing and participated in manuscript review. GD participated in the conduct of the study and the manuscript review. FG assisted with the study execution and critically reviewed the manuscript. FH participated in the design of the study, participated in the execution of the study and critically reviewed the manuscript. GK participated in the planning, methodological concepts, interpretation of results and manuscript review. AN participated in the conduct of the study and the manuscript review. UB designed the study, obtained funding, supervised and assisted with its execution, supervised analysis and interpretation of data, and critically reviewed the manuscript.

All authors confirmed that they have seen and approved the final version and have no conflicts of interest.

## Pre-publication history

The pre-publication history for this paper can be accessed here:

http://www.biomedcentral.com/1471-2334/10/8/prepub
